# Combined Application of Ultrasound and CT Increased Diagnostic Value in Female Patients with Pelvic Masses

**DOI:** 10.1155/2016/6146901

**Published:** 2016-10-27

**Authors:** Yan Liu, Hui Zhang, Xiaoqian Li, Guiqin Qi

**Affiliations:** ^1^Department of Ultrasound, The Hospital Affiliated to Taishan Medical University, 706 Taishan Avenue, Tai'an 271000, China; ^2^Department of Gynaecology, The Hospital Affiliated to Taishan Medical University, 706 Taishan Avenue, Tai'an 271000, China; ^3^Department of Radiology, The Hospital Affiliated to Taishan Medical University, 706 Taishan Avenue, Tai'an 271000, China; ^4^Department of Out-Patient, The Hospital Affiliated to Taishan Medical University, 706 Taishan Avenue, Tai'an 271000, China

## Abstract

*Purpose*. The current study aimed to evaluate whether combined application of ultrasound and CT had increased Diagnostic Value in Female Patients with Pelvic Masses over either method alone.* Patients and Methods*. 240 female patients with pelvic masses were detected preoperatively with ultrasound and CT prior to surgery. The sensitivity, specificity, and accuracy of ultrasound, CT, and combined ultrasound/CT application were evaluated, respectively.* Results*. The sensitivity, specificity, and accuracy of ultrasound were 52.8%, 86.7%, and 68.75%, respectively. The sensitivity, specificity, and accuracy of CT were 80.3%, 90.3%, and 85%, respectively. The sensitivity, specificity, and accuracy of combined application of ultrasound and CT were 89%, 94.7%, and 91.7%. The sensitivity, specificity, and accuracy of combined application of ultrasound and CT were higher than those of either ultrasound or CT.* Conclusions*. The combined application of ultrasound and CT had higher Diagnostic Value in Female Patients with Pelvic Masses than either method alone.

## 1. Introduction

It was reported that a pelvic mass will occur in about 20% of women at some time in their lives [1]. However, most women are unaware of the pelvic masses until they were diagnosed during routine physical or gynecologic examinations, and it is still difficult for gynecologists to diagnose and manage the pelvic masses.

Approximately 289,000 women in the United States are diagnosed with a pelvic mass each year [2]. When patients are diagnosed with pelvic masses, the first and most important thing is to determine the origin of the masses and then to evaluate whether they are benign or malignant. Although the majority of the pelvic mass was diagnosed with benign disease, 5–10% was diagnosed with an ovarian cancer, which is the fifth most common cause of cancer death in women worldwide [3–5]. As mortality was related to disease stage closely, an early diagnosis plays a vital role in determining a timely surgical and/or chemotherapeutic treatment [6]. Preoperative prediction of pelvic mass for gynecologists plays an important role in the treatment of patients with a pelvic mass. Various diagnostic methods involving morphological appearance, demographic data, vascular flow, and biomarkers, as well as molecular profiles, have been developed to distinguish malignant masses from benign masses; therefore improving methods would improve the accuracy of disease prediction [7].

A pelvic mass can be solid (fibroma), cystic (cystadenoma), or both (dermoid), and it can be benign or malignant. Different imaging methods have been used to predict the pelvic mass preoperatively. However, it is difficult for one single diagnostic method to predict disease with high accuracy, and a combination of parameters approach (CT and ultrasound) shows an ideal option. Yuan et al. reported that combined application of ultrasound and SPECT/CT has incremental value in detecting parathyroid tissue in SHPT patients; the sensitivity, specificity, and accuracy of combined application of ultrasound and ^99m^Tc-sestamibi SPECT/CT were higher than those of either ultrasound or ^99m^Tc-sestamibi SPECT/CT [8].

In current study, we try to investigate whether combined application of ultrasound and CT increased Diagnostic Value in Female Patients with Pelvic Masses over either method alone.

## 2. Materials and Methods

### 2.1. Patients

The study was approved by the ethics committee of Taishan Medical University. The written informed consent was obtained from the patients.

All female patients were women hospitalized in the Hospital Affiliated to Taishan Medical University who undergo laparoscopic surgery due to pelvic mass from July 2013 to July 2015.

The considering confidence level of 95% and accuracy of* d*: 0.01 were at least 150 cases. 240 cases were introduced in the study.

At first, we obtained the patients information including history and age, physical examination findings, sonographic and CT scan results, pathologic results (benign and malignant), and suggested procedure based on each mentioned method and surgical method carried out. All sonographic images were taken with Philips iU22 Ultrasound Systems (Philips Healthcare, Andover, MA, USA) and CT scan images were taken with light-speed plus (GE Company) apparatus, respectively, in this study.

The positive result by CT or ultrasound was defined as the true positive of combined application of ultrasound and CT, while the negative result by ultrasound and CT was defined as the true negative of combined application of ultrasound and CT.

### 2.2. Preoperative Planning

According to the ultrasound, CT, and pathological results, in the meantime, the surgeons did preoperative planning.

### 2.3. Statistical Analysis

All data was performed by SPSS 17.0 software (SPSS Inc., Chicago, IL, USA).

Comparisons between groups were performed using the chi-square test. A* P* value less than 0.05 was considered statistically significant.

## 3. Results

240 patients presented with pelvic mass were included in this study. All these patients were hospitalized in the Hospital Affiliated to Taishan Medical University for laparoscopic surgery. They aged from 18 to 77 years with the mean age of 41.16 ± 15.21. According to pathologic examinations from these 240 patients, 127 (52.9%) cases were malignant, while 113 (47.1%) cases were benign.

Masses that were diagnosed were shown in Table 4.

### 3.1. Ultrasound and CT Scan Results

Comparison of ultrasound, CT, combined ultrasound and CT, and pathological results were shown in Table 1.

The sensitivity, specificity, and accuracy of ultrasound were 52.8% (67/127), 86.7% (98/113), and 68.8% (165/240), respectively. The sensitivity, specificity, and accuracy of ^99m^Tc-sestamibi SPECT/CT were 80.3% (102/127), 90.3% (102/113), and 85.0% (204/240), respectively. The sensitivity, specificity, and accuracy of combined application of ultrasound and ^99m^Tc-sestamibi SPECT/CT were 89.0% (113/127), 94.7% (107/113), and 91.7% (220/240), respectively (Table 2).

### 3.2. Suggested Treatment

60% of cases should have mass removal according to ultrasound examination results, and 36.3% of CT scan cases should have mass removal, while this measure was adopted in 45% of combination of ultrasound and CT examination cases, which is more consistent with surgical method based on pathological examination results (47.1% mass removal) (shown in Table 3).

Hysterectomy was 5.4% in ultrasound cases and 5% in CT scan cases, and this method supposed by the combination examination and pathologic results was 3% and 1.7%, respectively (shown in Table 3).

Mass removal and staging were in 10.8% of ultrasound examination patients and 16.7% in CT scan cases, while the combination of ultrasound and CT examination is more efficient in accurate prediction (shown in Table 3).

Hysterectomy with oophorectomy without staging was 14.2% in ultrasound examination cases and 12.5% in CT scan cases, while the appropriate therapeutic measure based on combination method and pathologic results in 6.3% and 3.3% of patients was oophorectomy with staging, respectively (shown in Table 3).

The appropriate therapeutic measure based on ultrasound examination and CT scan results in 9.6% and 29.6% of patients was oophorectomy with staging, respectively, while this measure was taken in 32.1% of combination of ultrasound and CT examination cases, that is, more consistent with surgical method based on pathological examination results (34.6%) (shown in Table 3).

## 4. Discussion

A pelvic mass is a swelling or an enlargement in the pelvic region, which may originate from either the gynecologic organs (the uterus, cervix, and uterine adnexa) or other pelvic organs (the bladder, intestines, ureters, and renal organs) [9–11]. Most pelvic masses are benign conditions, such as an ovarian cyst, while others may be malignant [12, 13]. According to the American Cancer Society estimates for ovarian cancer in the United States in 2016, about 22,280 women will receive a new diagnosis of ovarian cancer, while about 14,240 women will die from ovarian cancer [14].

Early and proper therapy are important in decreasing the death, such as surgery, chemotherapy, hormone therapy, and targeted therapy, as well as radiation therapy [15, 16]. In order to have patients treated by optional way, a correct preoperative diagnosis of pelvic masses is very important. Physical exam, imaging tests like computed tomography (CT) scans, magnetic resonance imaging (MRI) scans, and ultrasound studies, and other tests may provide useful information about pelvic mass [17, 18]. However, it is very limited for one test to give an accurate diagnosis; for example, diagnostic ultrasound is only about 50% sensitive for stage I epithelial ovarian cancer and is further limited by poor specificity in accurately differentiating benign from malignant pathology [19].

In the present study, we evaluate whether combined application of ultrasound and CT had increased Diagnostic Value in Female Patients with Pelvic Masses over either method alone. The present study showed that the combined application of ultrasound and CT scan preoperatively had a higher sensitivity, specificity, and accuracy than those of ultrasound or CT scan alone. As ultrasound is noninvasive, is easily repeatable, and has acceptable sensitivity and specificity, it is used widely in routine examination for pelvic mass. Theodoridis et al. reported that ultrasound examination had lower sensitivity (50%) and specificity (92%) in the detection of ovarian borderline tumors [20]. Firoozabadi et al. reported that the sensitivity and specificity of sonography-physical examination were 51.9% and 87.9%, respectively, and the sensitivity and specificity of CT scan images were 79.2% and 91.6%, respectively [21]. In this study, the sensitivity, specificity, and accuracy of ultrasound were determined to be 52.8%, 86.7%, and 68.75%, respectively, and the sensitivity, specificity, and accuracy of CT scan were determined to be 80.3%, 90.3%, and 85%, respectively, which are significantly higher than those of ultrasound (*P* < 0.05). (Chi-square test was used, and the* P* value of sensitivity, specificity, and accuracy between two groups is 3.141023*e* − 005, 0.4, and 0.004, resp.) However, the sensitivity, specificity, and accuracy of combined application of ultrasound and CT were 89%, 94.7%, and 91.7%, which were higher than those of either ultrasound or CT. The Positive Predictive Values and Negative Predictive Values of ultrasound method are 81.7% and 62.0%, respectively. The Positive Predictive Values and Negative Predictive Values of CT scan method are 90.3% and 80.3%, respectively. The Positive Predictive Values and Negative Predictive Values of combined ultrasound + CT scan method are 95.0% and 88.4%, respectively. Yuan et al. also showed combined application of ultrasound and SPECT/CT has incremental value in detecting parathyroid tissue in SHPT patients [8].

Transabdominal and transvaginal ultrasound play an important role in the diagnosis of pelvic masses, and transvaginal sonography is superior to transabdominal sonography in most cases of pelvic masses. However, transabdominal ultrasound was still the initial sonographic technique for routine evaluation of the female pelvis. In current study, we applied transabdominal ultrasound in the evaluation of the female pelvis. Transabdominal sonography yielded variable results in evaluating pelvic masses, with a sensitivity of 50%–100% and a specificity of 46%–100% [22]. In current study the sensitivity, specificity, and accuracy of sonography were much less than those of known published data [20]. Low quality of the sonography devices and the level of sonographer's proficiency in our hospital might contribute to the results. For other reasons, varying threshold values and corresponding tradeoffs between sensitivity and specificity may result in different results partly [23, 24].

Furthermore, the combined application of ultrasound and CT is particularly complementary and useful in the planning of the surgical strategy of pelvic mass.

In most mentioned surgical methods (mass removal, oophorectomy with staging, mass removal with staging, and hysterectomy), prediction of surgical method based on combination of ultrasound and CT method had more consistency with appropriate surgical treatment based on pathologic results.

## 5. Conclusions

Our study demonstrates that the combined application of ultrasound and CT has incremental value in accurately detecting pelvic mass over either method alone.

## Figures and Tables

**Table 1:** Comparison of ultrasound, CT, combined ultrasound and CT, and pathological results.

	Ultrasound		CT		Combination method		Pathological	
	Number	Percent	Number	Percent	Number	Percent	Number	Percent
Benign	158	65.8%	127	52.9%	121	50.4%	113	47.1%
Malignant	82	34.2%	113	47.1%	119	49.6%	127	52.9%

Total	240	100%	240	100%	240	100%	240	100%

**Table 2:** Sensitivity, specificity, and accuracy of ultrasound, CT, and combined ultrasound and CT.

	Sensitivity	Specificity	Accuracy
Ultrasound	52.8%	86.7%	68.75%
CT	80.3%	90.3%	85.0%
Combined ultrasound and CT	89.0%	94.7%	91.7%

**Table 3:** Suggested treatment based on different tests.

Treatment	Ultrasound	CT	Combined application	Pathological
A	144 (60%)	87 (36.3%)	108 (45%)	113 (47.1%)
B	13 (5.4%)	12 (5%)	7 (3%)	4 (1.7%)
C	26 (10.8%)	40 (16.7%)	33 (13.8%)	32 (13.3%)
D	34 (14.2%)	30 (12.5%)	15 (6.3%)	8 (3.3%)
E	23 (9.6%)	71 (29.6%)	77 (32.1%)	83 (34.6%)

Total	240 (100%)	240 (100%)	240 (100%)	240 (100%)

A: mass removal; B: hysterectomy; C: mass removal and staging; D: hysterectomy with oophorectomy; E: oophorectomy with staging.

**Table 4:** The diagnosis of masses.

	Uterine fibroids	Endometrial cancer	Cervical cancer	Uterine sarcoma	Benign ovarian tumor	Ovarian cancer
Ultrasound	109	23	41	4	49	14
CT	87	29	58	7	40	19
Combination method	84	31	60	8	37	20
Pathological	78	33	64	9	35	21

## References

[1] Moore R. G., Bast R. C. (2007). How do you distinguish a malignant pelvic mass from a benign pelvic mass? Imaging, biomarkers, or none of the above. *Journal of Clinical Oncology*.

[2] Lokich E., Palisoul M., Romano N. (2015). Assessing the risk of ovarian malignancy algorithm for the conservative management of women with a pelvic mass. *Gynecologic Oncology*.

[3] Pejovic T., Nezhat F. (2001). Laparoscopic management of adnexal masses the opportunities and the risks. *Annals of the New York Academy of Sciences*.

[4] Jemal A., Siegel R., Ward E. (2008). Cancer statistics, 2008. *CA Cancer Journal for Clinicians*.

[5] Goh W., Bohrer J., Zalud I. (2014). Management of the adnexal mass in pregnancy. *Current Opinion in Obstetrics & Gynecology*.

[6] Sandri M. T., Bottari F., Franchi D. (2013). Comparison of HE4, CA125 and ROMA algorithm in women with a pelvic mass: correlation with pathological outcome. *Gynecologic Oncology*.

[7] Yanaranop M., Tiyayon J., Siricharoenthai S., Nakrangsee S., Thinkhamrop B. (2016). Rajavithi-ovarian cancer predictive score (R-OPS): a new scoring system for predicting ovarian malignancy in women presenting with a pelvic mass. *Gynecologic Oncology*.

[8] Yuan L., Kan Y., Ma D., Yang J. (2016). Combined application of ultrasound and SPECT/CT has incremental value in detecting parathyroid tissue in SHPT patients. *Diagnostic and Interventional Imaging*.

[9] Wakayama A., Inamine M., Kudaka W. (2013). Concurrent chemoradiotherapy for non-bulky stage IB/II cervical cancer without pelvic node enlargement. *Anticancer Research*.

[10] Shwayder J., Sakhel K. (2014). Imaging for uterine myomas and adenomyosis. *Journal of Minimally Invasive Gynecology*.

[11] Paltiel H. J., Phelps A. (2014). Us of the pediatric female pelvis. *Radiology*.

[12] Smorgick N., Maymon R. (2014). Assessment of adnexal masses using ultrasound: a practical review. *International Journal of Women's Health*.

[13] Geurts S. M. E., de Vegt F., van Altena A. M. (2011). Considering early detection of relapsed ovarian cancer: a review of the literature. *International Journal of Gynecological Cancer*.

[14] Siegel R. L., Miller K. D., Jemal A. (2016). Cancer statistics, 2016. *CA: Cancer Journal for Clinicians*.

[15] Cohen J. G., White M., Cruz A., Farias-Eisner R. (2014). In 2014, can we do better than CA125 in the early detection of ovarian cancer?. *World Journal of Biological Chemistry*.

[16] Ignacio E. A., Dua R., Sarin S. (2008). Pelvic congestion syndrome: diagnosis and treatment. *Seminars in Interventional Radiology*.

[17] Engelen M. J. A., Bongaerts A. H. H., Sluiter W. J. (2008). Distinguishing benign and malignant pelvic masses: the value of different diagnostic methods in everyday clinical practice. *European Journal of Obstetrics Gynecology and Reproductive Biology*.

[18] Tingulstad S., Hagen B., Skjeldestad F. E. (1996). Evaluation of a risk of malignancy index based on serum CA125, ultrasound findings and menopausal status in the pre-operative diagnosis of pelvic masses. *British Journal of Obstetrics and Gynaecology*.

[19] Cohen L. S. (2008). *Diagnostic Ultrasound in the Assessment of the Adnexal Mass*.

[20] Theodoridis T. D., Zepiridis L., Mikos T. (2009). Comparison of diagnostic accuracy of transvaginal ultrasound with laparoscopy in the management of patients with adnexal masses. *Archives of Gynecology and Obstetrics*.

[21] Firoozabadi R. D., Zarchi M. K., Mansurian H. R., Moghadam B. R., Teimoori S., Naseri A. (2011). Evaluation of diagnostic value of CT scan, physical examination and ultrasound based on pathological findings in patients with pelvic masses. *Asian Pacific Journal of Cancer Prevention*.

[22] Yong-Yeon Jeong E. K. O., Kang H. K. (2000). Imaging evaluation of ovarian masses. *RadioGraphics*.

[23] Khan S. A., Banoo A. (2014). Role of colour doppler sonography in adnexal masses. *International Journal of Medical Research & Health Sciences*.

[24] Gupta N. (2013). Adnexal masses in perimenopausal women: how effective is color flow mapping and pulse doppler waveform studies in detecting malignancy preoperatively?. *The Journal of South Asian Federation of Menopause Societies*.

